# The Portsmouth Meeting of the British Medical Association

**Published:** 1899-07-29

**Authors:** 


### THE PORTSMOUTH MEETING OF THE BRITISH MEDICAL ASSOCIATION.

The meeting of the British Medical Association, which will assemble next week at Portsmouth, is likely to be a very successful one. It has everything in its favour. It is held in a large and important town, which is also a centre from which many charming excursions can be made, besides being a great naval and military rendezvous, and its president, Dr. John Ward Cousins, senior surgeon to the Royal Portsmouth Hospital, is not only well known and highly respected in the surgical world, but has for years been honourably connected with much of the best work of the association, and from the regularity with which he has attended the annual meetings, he has become personally well known to many among that curiously-assorted collection of all sorts and conditions of medical men who take delight in these semi-social, semi-scientific gatherings. Naturally, a meeting which is to take place in a town so intimately concerned with naval and military affairs, will have in its programme a fairly strong dash of the services. And most appropriately so. The naval officers may not have received of late, perhaps may not have required, any special assistance from the outside profession as viewed by the association; but the Army Medical Department, kept in silence as of necessity it has been by the rules of the service, has without doubt been much indebted to the active intervention of the association for much of improved status which its officers now enjoy, and for the removal of many of the grievances which for so many years kept the service under a cloud. Among the discussions of naval and military interest which are promised at the coming meeting we find the following: On the medical tests required at present for admission to the public services; on the diagnosis and treatment of gunshot wounds of the abdomen; on the prevention of syphilis in the navy and army; and on the visual tests employed in the navy, army, and mercantile marine. Of other subjects, however, there is no lack; indeed, our wonder is excited at the boldness shown by the organisers of the feast in expecting their visitors to discuss, never mention to digest, the enormous menu which they have drawn up. Tuberculosis crops up in many phases, and as the discussion on its preventive and remedial treatment is to be opened by Professor Clifford Allbutt we may expect to see the whole question displayed with that open-minded reasonableness of which he is so well known an exponent. In few things is there at the present moment greater danger of being dragged at the heel of imperious fashion to unsound conclusions than in regard to tuberculosis and its prevention, and we are glad to see the names of some very sane persons among those who are going to take part in the discussion. For those who do not care about tuberculosis, gout will be served up in many forms, and uric acid, which in some hands is quite a *sauce piquante*, will be considered in its various relations. Among other things in the same section will be a paper by Dr. Ewart on some clinical uses of percussion, and if it should happen that he should give a demonstration on the subject, it will no doubt be worth attending, as showing what percussion can be made to do. In surgery appendicitis as usual crops up, and some good cases of operative treatment in malignant disease of the stomach will be discussed. This is a matter which deserves the most careful consideration by the many general practitioners usually present at these meetings. There clearly is a great future for these operations if the cases can be got hold of early enough. In the same section the demonstration of X-Ray photography will no doubt prove attractive. In the obstetrical section another of what we may call the dangerous and critical subjects, but one also of wide practical interest, is to be discussed, namely, the use of serumtherapy in the treatment of fever following delivery. The discussion will be opened by Dr. Herbert Spencer, who we have no doubt will handle it carefully. The section of State medicine will discuss vaccination, tuberculosis, and "school influence" in the spread of infection, and as for the subjects to be dealt with in the other sections they are too numerous to mention. There can be no doubt, however, that what with the addresses, the sections, the entertainments, the garden parties, the excursions, and the meeting of old friends, he must indeed be hard to please who does not derive both pleasure and instruction from a visit to Portsmouth next week. We only hope the general meetings will behave themselves. Perhaps it might really be better if these were held at the end instead of at the beginning of the proceedings. When a few hundred doctors, just let out of harness, meet together, they are apt to be just a trifle obstreperous at first.

